# Comparison of the decompressive effect of different surgical procedures for dysthyroid optic neuropathy using 3D printed models

**DOI:** 10.1007/s00417-022-05645-2

**Published:** 2022-04-08

**Authors:** Kinga Yo, Kunihiro Nishimura, Yasuhiro Takahashi, Hiroki Yokota, Naoyuki Hatayama, Tetsuro Hoshino, Munekazu Naito, Tetsuya Ogawa, Yasushi Fujimoto

**Affiliations:** 1 Department of Otorhinolaryngology, Aichi Medical University School of Medicine, 1-1 Yazakokarimata, Nagakute, Aichi 4801195 Japan; 2 Nishimura ENT & Skin Clinic, Nagakute, Aichi Japan; 3 Department of Oculoplastic, Orbital and Lacrimal Surgery, Aichi Medical University Hospital, Nagakute, Aichi Japan; 4 Department of Mechanical Engineering, Meijo University, Nagoya, Aichi Japan; 5 Department of Anatomy, Aichi Medical University School of Medicine, Nagakute, Aichi Japan; 6 Department of Sleep Medicine and Sleep Disorder Center, Aichi Medical University Hospital, Nagakute, Aichi Japan; 7 Hoshino ENT and Sleep Disordered Breathing Center, Hyogo, Japan

**Keywords:** Medial orbital wall decompression, Inferomedial orbital wall decompression, Balanced orbital decompression, 3D printer, Dysthyroid optic neuropathy

## Abstract

**Purpose:**

To compare the decompressive effect around the optic nerve canal among 3 different decompression procedures (medial, balanced, and inferomedial) using 3D printed models.

**Methods:**

In this experimental study, based on data obtained from 9 patients (18 sides) with dysthyroid optic neuropathy, a preoperative control model and 3 plaster decompression models were created using a 3D printer (total, 72 sides of 36 models). A pressure sensor was placed at the optic foramen, and the orbital space was filled with silicone. The surface of the silicone was pushed down directly, and changes in pressure were recorded at 2-mm increments of pushing.

**Results:**

At 10 mm of pushing, there was significantly lower pressure in the medial (19,782.2 ± 4319.9 Pa, *P* = 0.001), balanced (19,448.3 ± 3767.4 Pa, *P* = 0.003), and inferomedial (15,855.8 ± 4000.7 Pa, *P* < 0.001) decompression models than in the control model (25,217.8 ± 6087.5 Pa). Overall, the statistical results for each 2-mm push were similar among the models up to 10 mm of pushing (*P* < 0.050). At each push, inferomedial decompression caused the greatest reduction in pressure (*P* < 0.050), whereas there was no significant difference in pressure between the medial and balanced decompression models *(P* > 0.050).

**Conclusion:**

All 3 commonly performed decompression procedures significantly reduced retrobulbar pressure. Because inferomedial decompression models obtained the greatest reduction in pressure on the optic nerve canal, inferomedial decompression should be considered the most reliable procedure for rescuing vision in dysthyroid optic neuropathy.


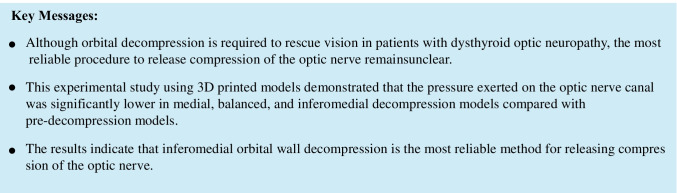


## Introduction

Dysthyroid optic neuropathy (DON) is the most severe manifestation of thyroid eye disease and causes irreversible vision loss [1]. In DON, enlarged extraocular muscles directly compress the optic nerve at the orbital apex, and the increased retrobulbar pressure reduces blood flow to the optic nerve [1]. Patients in whom DON is refractory to anti-inflammatory treatment require orbital decompression to release compression of the optic nerve and to reduce retrobulbar pressure [2].

Orbital decompression procedures are classified as bony or orbital fat decompression, and bony decompression is sub-classified further into the orbital roof, orbital floor, medial orbital wall (medial decompression), and deep lateral orbital wall (deep lateral decompression) [3]. Surgeons choose the type of procedure for the treatment of disfiguring proptosis according to the risk of postoperative diplopia, the severity of proptosis, and the surgeon’s preference [3]. However, when treating DON, the medial orbital wall procedure is usually performed because of the anatomical proximity between the medial orbital wall and optic canal [4, 5] and is sometimes combined with the orbital floor (inferomedial) or deep lateral (balanced) decompression [3, 4, 6–8]. To our knowledge, no study has investigated whether the decompressive effect on the optic nerve at the orbital apex varies according to the orbital decompression method used. The ability to select the optimal decompression procedure for DON would enable surgeons to save patients’ vision more reliably.

The recent development of 3D printers has enabled the creation of personalized anatomical models [9]. The purpose of this study was to compare the decompressive effect around the optic canal among medial decompression, inferomedial decompression, and balanced decompression using orbital models of patients with DON created by 3D printing.

## Materials and methods

### Patients and study design

We retrospectively reviewed the medical records of 9 consecutive patients who underwent orbital decompression for DON at Aichi Medical University Hospital between August 2012 and January 2018. Patients with missing clinical data were excluded. DON was diagnosed if optic nerve compression was seen on imaging in the absence of another cause of vision loss, and at least one of the following were present: decimal visual acuity of 0.5 or less, central flicker frequency of 35 Hz or less, positive relative afferent pupillary defect, central or paracentral scotoma, and papilledema or a pale optic disc [10]. The study was carried out as follows: First, we created a 3D plaster pre-decompression (control) model and 3 different decompression models (medial, balanced, and inferomedial) using preoperative computed tomographic (CT) data. Then, we measured the pressure on the optic nerve canal in each model and compared the results among the models to determine the decompression effect of each procedure.

### Data collection

Data on age, sex, previous surgical decompression procedures, preoperative clinical activity score, visual acuity, central flicker frequency, and Hertel exophthalmometric values were collected from the medical charts. Decimal visual acuity was converted to the logarithm of the minimum angle of resolution (logMAR). As in a previous report [11], visual acuity of counting fingers, hand motion, light perception, and no light perception were converted to logMAR of 2.6, 2.9, 3.1, and 3.4, respectively. Clinical activity scores and the Hertel exophthalmometry measurements were obtained by an oculoplastic specialist (Y.T.).

### Creation of 3D printed models

Contiguous 1-mm axial preoperative computed tomographic (CT) images (Aquilion Precision; Canon Medical System Corporation, Tochigi, Japan) were obtained with a bone window algorithm (width, 2500; level, 500). Axial CT images were obtained based on Reid’s baseline, with the reconstruction of coronal, sagittal, and 3D images. To obtain symmetric axial images, the positions of the face, shoulders, and soles of the feet were fixed with no bend in the patient’s neck. When symmetric axial images could not be obtained, the images were reconstructed to achieve symmetry.

**Fig. 1:**
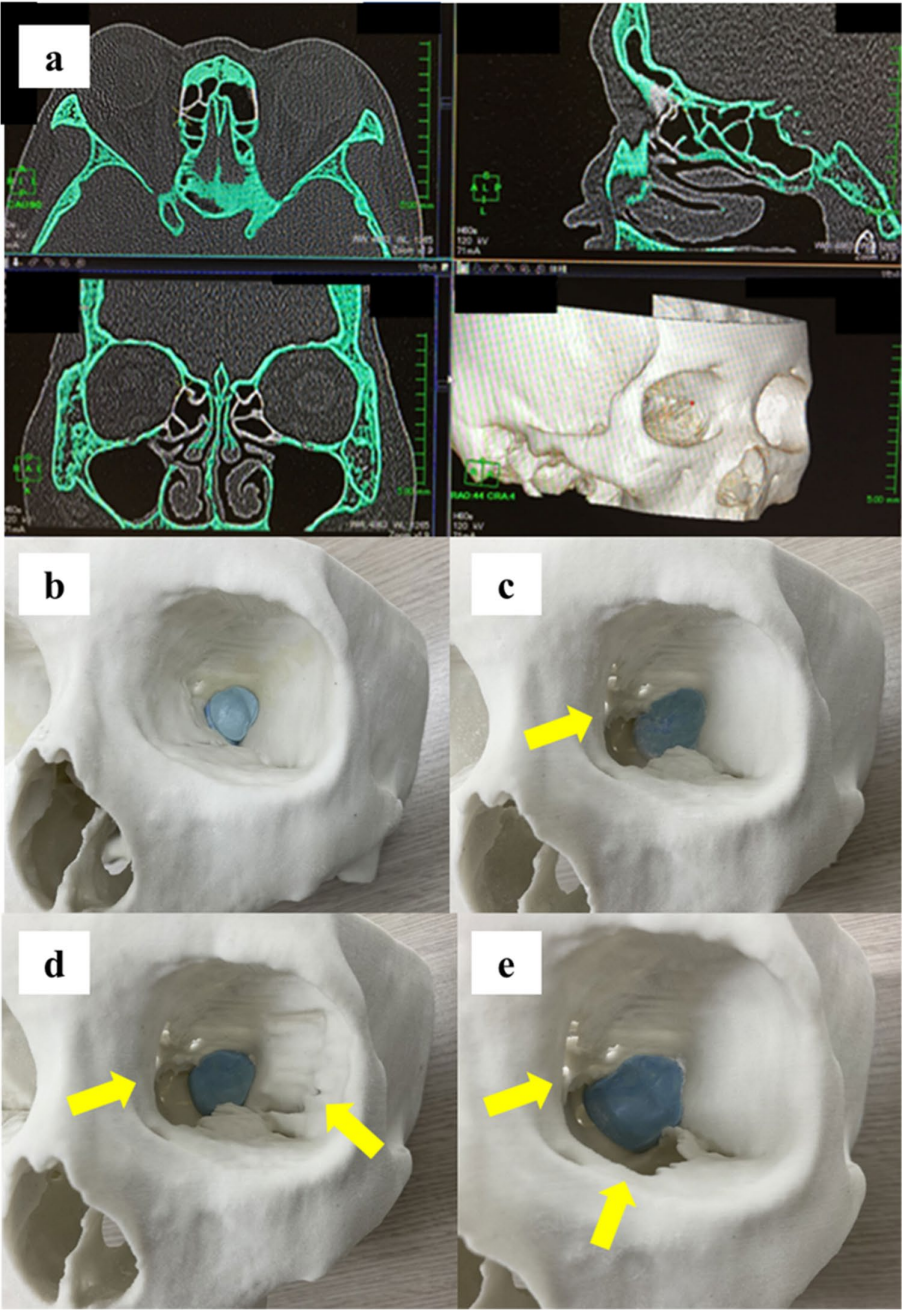
Creation of the 3D printed models. **a** Three types of decompression surgery were simulated using 3D image creation software. **b–e** The optic canal of each 3D printed model was puttied up. Arrows indicate the areas of bone removal. **b** A pre-decompression model was used as a control. **c** A medial decompression model. **d** A balanced decompression model. **e** An inferomedial decompression model

Preoperative CT data were entered into 3D image creation software (Virtual Place; Canon Medical System Corporation, Tochigi, Japan). Each decompression procedure (medial, inferomedial, and balanced) was simulated using the same software (Fig. 1a). For medial decompression, the upper limit of bone removal was the level of the frontoethmoidal suture, the lower limit was the level of the junction of the medial orbital wall and orbital floor, the anterior limit was the level a few millimeters below the posterior lacrimal crest, and the posterior limit was the level just above the sphenoid sinus. For orbital floor decompression, the anterior limit was the level a few millimeters posterior to the inferior orbital rim, the posterior limit was the level just anterior to the pterygopalatine fossa, the medial limit was the level of the junction of the medial orbital wall and the orbital floor, and the lateral limit was the infraorbital canal. For deep lateral orbital wall decompression, the greater wing of the sphenoid bone (the trigone) was removed. Plaster models of the preoperative (control) orbit and each postoperative orbit were created using a 3D printer (ProJet 160; 3D Systems, Rockhill, SC) (Fig. 1b−e).

### Measurements of pressure at the optic foramen

**Fig. 2:**
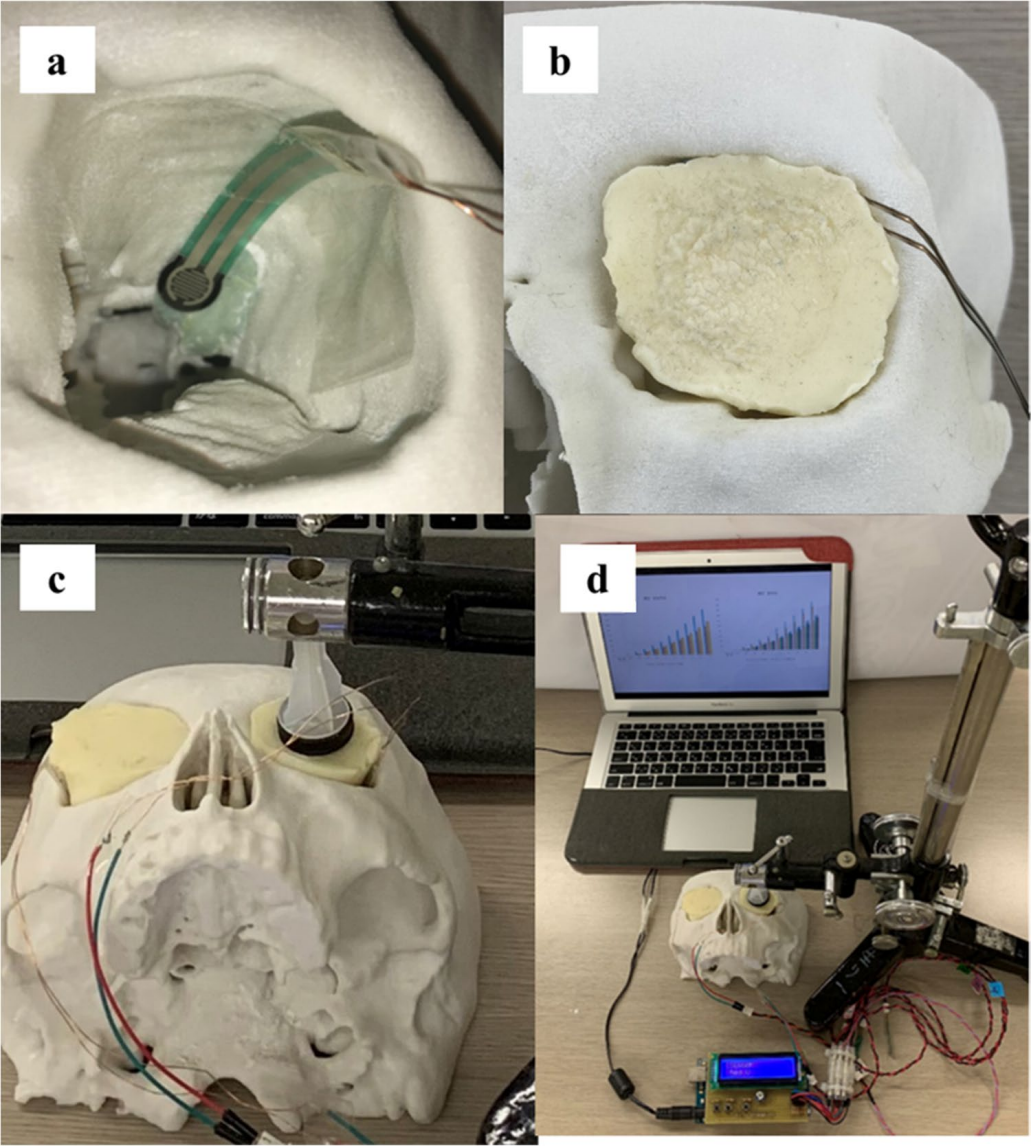
Pressure measurements at the optic nerve foramen. **a** Radial pressure sensor was placed on the putty that filled the optic canal. **b** The orbit was filled with silicone. **c** The gasket of an injection syringe was set over the silicone. **d** The silicone surface was pushed down and changes in pressure were recorded at 2-mm increments of pushing

The optic canal of all 3 models was puttied up (Exafine Putty Type, GC Corporation, Tokyo, Japan), and a pressure sensor (FSR400; Interlink Electronics, Inc., Camarillo, CA) was placed on the putty (Fig. 2a). The orbit was then filled with silicone (International Rubber Hardness Degree, 0) up to the level of the zygomaticofrontal suture (Fig. 2b), using an injection syringe. The silicone surface was pushed down directly at the intersection of the long and short axes using the gasket of a Terumo injection syringe (Terumo Corporation, Tokyo, Japan; volume, 20 mL; inner diameter, 21.7 mm; Fig. 2c). The gasket position was defined as 0 mm when it touched the surface of the silicone. The change in pressure at the optic foramen was then recorded at 2-mm increments of pushing the syringe for each model (Fig. 2d). The measurements were performed 3 times by one of the authors (K.Y.), and the mean values were used in the analysis.

### Evaluation of preoperative orbital status

Normally, the medial orbital wall has a laterally convex shape in the middle to a lower orbit, and the orbital floor has an S-shape in the anteroposterior direction with a superior bulge in the posterior portion [12]. However, these convex portions can be spontaneously decompressed by high retrobulbar pressure, particularly in patients with DON [12]. The effect of surgical decompression is suspected to be lower in cases with spontaneous decompression. Furthermore, the trigone is the main portion of bone removed during deep lateral decompression. The position and size of the trigone vary among individuals [13, 14], and the decompressive effect on the optic nerve is thought to be lower in cases with an anteriorly located trigone. Therefore, we evaluated the shapes of the medial orbital wall and orbital floor and the position of the trigone using preoperative axial and quasi-sagittal CT images.

**Fig. 3:**
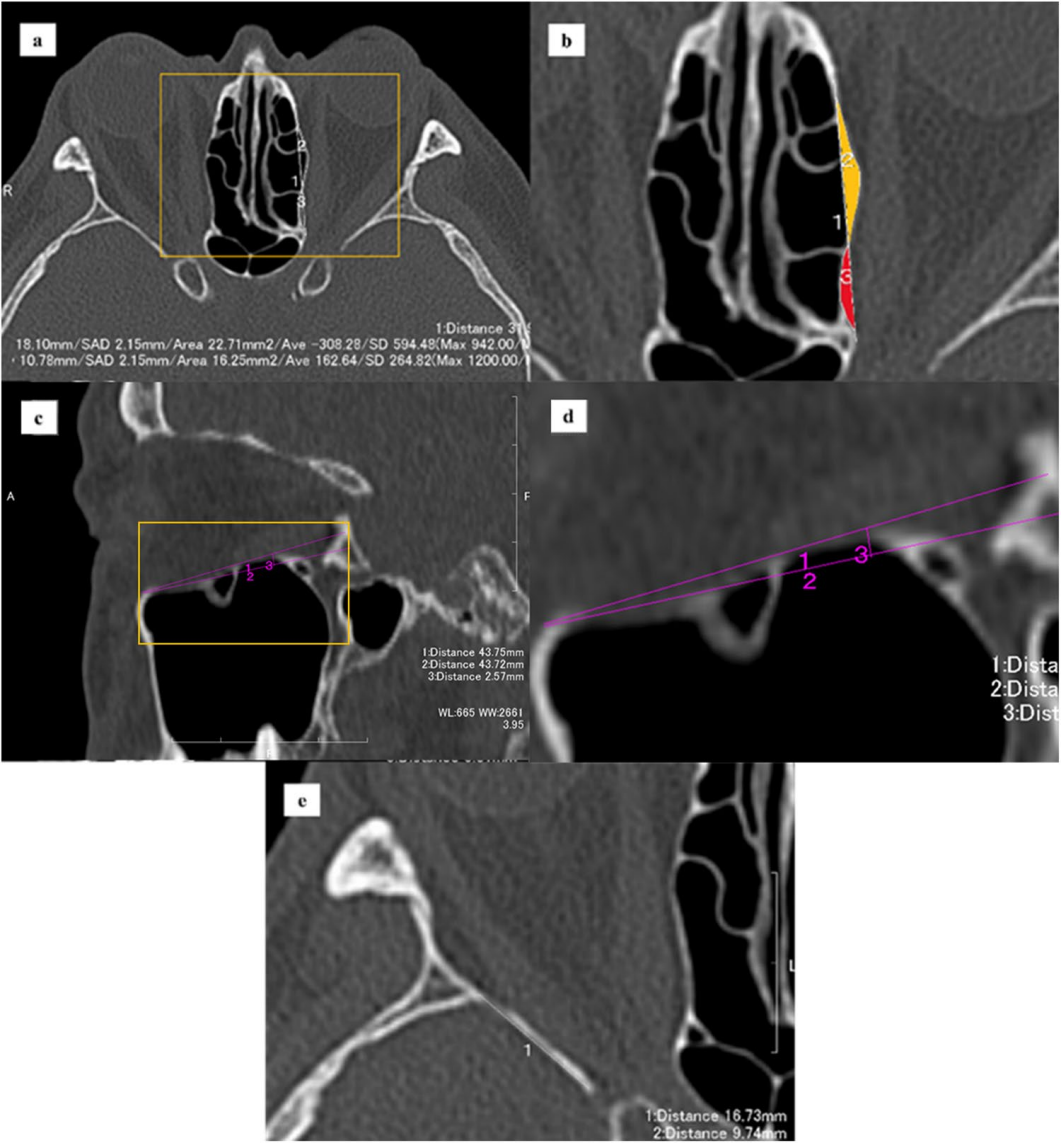
Evaluation of orbital wall configuration. **a**, **b** Axial computed tomography (CT) images showing the maximum cross-sectional area of the medial rectus muscle and bulged (yellow area in **b**) and dented (red area in **b**) areas in the medial orbital wall. **b** is a magnification of the rectangular portion in (**a**). **c**, **d** A line was drawn on the quasi-sagittal CT image through the optic nerve to connect the inferior orbital rim and the orbital process of the palatal bone. The length of the perpendicular distance between that line and the tip of the superior bulge of the orbital floor (length of the superior bulge) was then measured. **d** is a magnification of the rectangular portion in (**c**). **e** The distance between the posteromedial vertex of the trigone and the superior orbital fissure was measured

The shapes of the medial orbital wall and orbital floor were evaluated using the methods that we have reported previously [12]. On the axial CT image showing the maximum cross-sectional area of the medial rectus muscle, a line was drawn between the posterior lacrimal crest and the junction between the ethmoid bone and *corpus ossis sphenoidalis*, and the area that was bulged (convex pattern, expressed as a positive value) or dented (concave pattern, expressed as a negative value) from that line was measured (Fig. 3a and b). When the medial orbital wall contained both bulged and dented areas (mixed pattern), these areas were added.

On the quasi-sagittal CT image through the optic nerve, a line was drawn between the inferior orbital rim and the orbital process of the palatine bone; the length of the perpendicular distance from that line to the tip of the superior bulge of the orbital floor (length of the superior bulge) was measured (Fig. 3c and d), as in our previous study [12]. The length of the superior bulge was expressed as a positive value when the orbital floor bulged in a superior direction.

To evaluate the position of the trigone, the distance between the posteromedial vertex of the trigone and the lateral edge of the superior orbital fissure was measured on the axial CT image showing the maximum cross-sectional area of the lateral rectus muscle (Fig. 3e).

All measurements were performed by one of the authors (K.Y.) using digital calipers and freehand measurement tools available in image viewing software (ShadeQuest/ViewR; Yokogawa Medical Solutions Corporation, Tokyo, Japan).

### Statistical analysis

All values are shown as the mean ± standard deviation. The measurements of pressure at the optic foramen were compared for each model using one-way analysis of variance with Tukey–Kramer post hoc correction for multiple comparisons.

To evaluate the influence of the configuration of each orbital wall on orbital decompression, we analyzed the correlation between the pressures measured at the optic foramen and the results measured on preoperative CT images using Pearson’s correlation coefficient. We calculated the differences in pressure for each model by subtracting the pressure measured in the medial decompression model from that measured in the control for analysis of the medial orbital wall, from that measured in the balanced decompression model for analysis of the deep lateral orbital wall, and from that measured in the inferomedial decompression model for analysis of the orbital floor.

All statistical analyses were performed using SPSS version 22.0 software (IBM Corp., Armonk, NY). *P*-values < 0.05 were considered statistically significant.

## Results

The characteristics of the 9 patients with DON are shown in Table 1. Based on the CT data obtained for these patients, 36 3D models were created (72 sides; 18 sides in each group).

**Table 1:** Patient demographics, preoperative ophthalmic status, and surgical procedures performed

Patients (*n*)	9
Sex (male/female)	0/9
Age (years)	63.4 ± 13.9
Sides with DON (*n*)	15
Surgical decompression procedures	
Medial	12
Balanced	3
Preoperative clinical activity score	1.1 ± 1.1
Preoperative logMAR visual acuity (*n* = 15)	0.43 ± 0.47
Preoperative central flicker frequency (Hz) (*n* = 15)	23.1 ± 7.6
Preoperative Hertel exophthalmometric value (mm; *n* = 18)	20.5 ± 2.0

*DON*, dysthyroid optic neuropathy; *logMAR*, logarithm of the minimum angle of resolution

Results for the preoperative configuration of the orbital walls are shown in Table 2. Convex and mixed patterns were found on the medial orbital wall on 5 and 13 sides, respectively. None of the medial orbital walls showed a concave pattern. Similarly, none of the orbital floors showed spontaneous decompression with a negative value for the length of the superior bulge.

**Table 2:** Measurement results from preoperative computed tomographic images

Medial orbital wall pattern	
Convex	5
Mixed	13
Concave	0
Medial orbital wall area (mm^2^)	28.8 ± 19.5
Spontaneous orbital floor decompression	0
Distance of superior bulge (mm)	5.1 ± 1.6
Distance of posteromedial vertex of the trigone (mm)	11.6 ± 3.1

The pressures measured at the optic foramen for each decompression model according to the amount of pushing are shown in Table 3, and the results of the statistical analysis of differences in these pressures are shown in Table 4. Overall, there was no significant difference in the pressure values according to the amount of pushing. At 10 mm of pushing, there was significantly lower pressure in the medial (19,782.2 ± 4319.9 Pa, *P* = 0.001), balanced (19,448.3 ± 3767.4 Pa, *P* = 0.003), and inferomedial (15,855.8 ± 4000.7 Pa, *P* < 0.001) decompression models than in the control model (25,217.8 ± 6087.5 Pa). For each increment of pushing, the decompressive effect was greatest with inferomedial decompression (*P* < 0.050). Furthermore, the decompressive effect of the medial decompression model was similar to that of the balanced decompression model (*P* > 0.050).

**Table 3:** Pressures measured for each decompression model

Amount of pushing	Pressure (Pa)			
	Control	Medial decompression	Balanced decompression	Inferomedial decompression
2 mm	2483.1 ± 744.1	1543.9 ± 617.0	1662.1 ± 575.7	1866.1 ± 2992.3
4 mm	7267.0 ± 2067.4	5104.2 ± 1429.3	4763.2 ± 1332.6	3740.2 ± 1089.4
6 mm	12,759.1 ± 3467.1	9623.0 ± 2305.3	8927.6 ± 1877.4	7265.6 ± 2010.0
8 mm	19,366.9 ± 3965.2	14,544.7 ± 3088.1	13,827.8 ± 2958.2	11,252.9 ± 3270.0
10 mm	25,217.8 ± 6087.5	19,782.2 ± 4319.9	19,448.3 ± 3767.4	15,855.8 ± 4000.7

**Table 4:** Statistical comparison of pressures measured between the 3 decompression models

*P*-values		Amount of pushing				
		2 mm	4 mm	6 mm	8 mm	10 mm
Versus control	Medial decompression	0.004	0.004	0.004	0.001	0.001
	Balanced decompression	0.014	0.001	0.001	0.005	0.003
	Inferomedial decompression	< 0.001	< 0.001	< 0.001	< 0.001	< 0.001
Versus medial decompression	Balanced decompression	0.653	0.302	0.302	0.206	0.623
	Inferomedial decompression	0.003	0.006	0.006	0.001	0.002
Versus inferomedial decompression	Balanced decompression	0.001	0.002	0.002	0.005	< 0.001

Given the lack of a significant difference in the pressure measurements according to the amount of pushing, the measurements for the 10-mm push were used for correlation analyses. A minimal relationship was found between the effect of medial decompression and the configuration of the medial orbital wall (correlation coefficient, 0.180; *P* = 0.475) and between the effect of floor decompression and the configuration of the orbital floor (correlation coefficient, 0.132; *P* = 0.603). There was a substantial negative correlation between the effect of deep lateral decompression and the distance of the posteromedial vertex of the trigone from the superior orbital fissure (correlation coefficient, − 0.640; *P* = 0.004).

## Discussion

This is the first study to compare the pressure at the optic foramen among 3 orbital decompression procedures using 3D printed models created based on high-precision CT data obtained from patients with DON. Although all decompression models had a significant decompressive effect in comparison with the control model, the effect was greatest for inferomedial decompression. Release of optic nerve compression and reduction of retrobulbar pressure by orbital decompression surgery is essential in patients with DON to avoid irreversible vision loss. The results of this study suggest that inferomedial decompression is best able to rescue vision.

Medial decompression is commonly used to treat DON because of the anatomical proximity between the medial orbital wall and the optic canal [4, 5]. In contrast, the orbital floor has less proximity to the optic canal because of the interposition of the pterygopalatine fossa between these structures. However, in this study, additional orbital floor decompression had a further decompressive effect on the optic canal compared with the medial decompression alone. The orbital floor appears as an S-shape in the anteroposterior direction and shows a superior bulge in the posterior portion [12]. This anatomical feature causes high retrobulbar pressure in the posterior orbit. Therefore, removal of the orbital floor, including the posterior portion, may have a sufficient decompressive effect on the optic nerve at the orbital apex.

The configurations of the medial orbital wall and orbital floor were not correlated with the effect of decompression surgery on the orbital walls. These results were unexpected in view of our hypothesis that the effect of surgical decompression would be lower in cases with spontaneous decompression and indicate that pressure on the optic nerve can be released irrespective of the configuration of the medial orbital wall and orbital floor.

Inferomedial decompression is associated with a high incidence of postoperative diplopia because it disrupts the alignment of the courses of the medial and inferior rectus muscles [3, 5, 15]. These extraocular muscles are often markedly enlarged in patients with DON, and misalignment of these enlarged muscles further increases the risk of new-onset diplopia after surgery [16]. Surgeons should inform patients with DON about this possible complication and its management in advance of surgery.

We found no significant difference in the pressure at the optic foramen between the medial and balanced decompression methods. This finding is in contrast with the notion that the decompressive effect is improved by increasing the number of orbital walls removed [15]. Anatomically, the superior orbital fissure is located between the lateral orbital wall and the optic canal, and the posterior border of the trigone sometimes bows [14]. These features may lessen the decompressive effect at the orbital apex after deep lateral decompression. Moreover, in this study, we found a substantial negative correlation between the effect of deep lateral decompression and the distance of the posteromedial vertex of the trigone from the superior orbital fissure. This finding implies that less reduction in pressure on the optic nerve is achieved in patients with a more anteriorly located trigone.

Given that patients with DON usually have mild proptosis [17], they did not need much retro-placement of the globe. Furthermore, deep lateral decompression poses the risks of cerebrospinal fluid leak, hypoesthesia in the temporal, cheek, and frontal regions, decreased tear secretion, oscillopsia, and temporal muscle wasting [3]. Moreover, the removal of multiple orbital walls increases the risk of new-onset diplopia after surgery [14]. Avoidance of deep lateral orbital wall removal may help to obtain an adequate decompressive effect with less risk of postoperative complications, especially in patients with an anteriorly located deep lateral orbital wall. However, combined deep lateral decompression may be indicated for patients with severe swelling of the lateral rectus muscle.

Although comparison of the decompressive effect on the optic nerve between the different orbital decompression procedures in patients with DON would provide more accurate results, this would be difficult for ethical reasons. Fortunately, the recent advent of techniques for 3D data acquisition, modeling, and printing has made it possible to create physical models for research and education purposes. Furthermore, 3D printed models are now entering clinical use. In the field of oculoplastic surgery, for example, they are being used for orbital fracture reduction and orbital brachytherapy and to create orbital prostheses [9, 18, 19]. Elaborate personalized 3D printed models can be reproduced and processed freely, as with the different decompression models created in our study. Our results suggest that 3D printed models will be suitable for use in patients with DON.

We measured the pressure at the optic foramen in an experimental system at 2, 4, 6, 8, and 10 mm of pushing, which might not reflect actual retrobulbar pressure. However, considering that the results for each increment of pushing were similar, we believe that our results are applicable to the management of DON in patients. However, in DON, orbital pressure is increased internally by the expansion of the orbital contents and externally by a tight eyelid and the orbital septum pressing down on the proptotic eye [20, 21]. Although our study method simulated the external force, we did not measure the internal force. Hence, future studies are necessary to evaluate changes in the internal force in patients with DON.

This study had several limitations. First, it included a small number of patients. Therefore, it may not have been adequately powered to detect statistically significant differences in decompressive effect between the different decompression models. Second, the patients included in this study were all Japanese. Considering the known racial variations in orbital anatomy [22], our results might not be applicable to other races. Third, the mean age in our case series was 63.4 ± 13.9 years, which seems to be higher than that of patients with thyroid eye disease without DON [23]. However, because DON commonly develops in older patients, the age range of the patients included in this study was appropriate [24]. Fourth, the models used in this study could not reproduce the orbital contents, including the extraocular muscles, orbital fat, nerves, and vessels. Instead, the bony structures were made of plaster, which did not sufficiently reproduce the hardness and rigidity of bone. However, it is important to note that we still found significant differences in the measurements obtained by high-precision CT and the 3D printed models.

In conclusion, this experimental study using 3D printed models demonstrated that the inferomedial decompression method had the greatest decompressive effect at the optic foramen. Although the medial and balanced decompression methods also significantly reduced the pressure at the optic foramen, there was no difference in effect between these procedures, especially in patients with an anteriorly located trigone. The results of this study suggest that although surgeons should consider the severity of DON, anatomical variation of the orbit, and risk of complications, inferomedial decompression is the most reliable procedure for release of optic nerve compression and reduction of retrobulbar pressure.
